# Swimming eukaryotic microorganisms exhibit a universal speed distribution

**DOI:** 10.7554/eLife.44907

**Published:** 2019-07-16

**Authors:** Maciej Lisicki, Marcos F Velho Rodrigues, Raymond E Goldstein, Eric Lauga

**Affiliations:** 1 Department of Applied Mathematics and Theoretical PhysicsUniversity of CambridgeCambridgeUnited Kingdom; 2 Institute of Theoretical Physics, Faculty of PhysicsUniversity of WarsawWarsawPoland; Massachusetts Institute of TechnologyUnited States; Massachusetts Institute of TechnologyUnited States

**Keywords:** ciliates, flagellates, log-normal distribution, Other

## Abstract

One approach to quantifying biological diversity consists of characterizing the statistical distribution of specific properties of a taxonomic group or habitat. Microorganisms living in fluid environments, and for whom motility is key, exploit propulsion resulting from a rich variety of shapes, forms, and swimming strategies. Here, we explore the variability of swimming speed for unicellular eukaryotes based on published data. The data naturally partitions into that from flagellates (with a small number of flagella) and from ciliates (with tens or more). Despite the morphological and size differences between these groups, each of the two probability distributions of swimming speed are accurately represented by log-normal distributions, with good agreement holding even to fourth moments. Scaling of the distributions by a characteristic speed for each data set leads to a collapse onto an apparently universal distribution. These results suggest a universal way for ecological niches to be populated by abundant microorganisms.

## Introduction

Unicellular eukaryotes comprise a vast, diverse group of organisms that covers virtually all environments and habitats, displaying a menagerie of shapes and forms. Hundreds of species of the ciliate genus *Paramecium* (Wichterman, 1986) or flagellated *Euglena* (Buetow, 2011) are found in marine, brackish, and freshwater reservoirs; the green algae *Chlamydomonas* is distributed in soil and fresh water world-wide (Harris et al., 2009); parasites from the genus *Giardia* colonize intestines of several vertebrates (Adam, 2001). One of the shared features of these organisms is their motility, crucial for nutrient acquisition and avoidance of danger (Bray, 2001). In the process of evolution, single-celled organisms have developed in a variety of directions, and thus their rich morphology results in a large spectrum of swimming modes (Cappuccinelli, 1980).

Many swimming eukaryotes actuate tail-like appendages called flagella or cilia in order to generate the required thrust (Sleigh, 1975). This is achieved by actively generating deformations along the flagellum, giving rise to a complex waveform. The flagellar axoneme itself is a bundle of nine pairs of microtubule doublets surrounding two central microtubules, termed the '9 + 2' structure (Nicastro et al., 2005), and cross-linking dynein motors, powered by ATP hydrolysis, perform mechanical work by promoting the relative sliding of filaments, resulting in bending deformations.

Although eukaryotic flagella exhibit a diversity of forms and functions (Moran et al., 2014), two large families, ‘flagellates’ and ‘ciliates’, can be distinguished by the shape and beating pattern of their flagella. Flagellates typically have a small number of long flagella distributed along the bodies, and they actuate them to generate thrust. The set of observed movement sequences includes planar undulatory waves and traveling helical waves, either from the base to the tip, or in the opposite direction (Jahn and Votta, 1972; Brennen and Winet, 1977). Flagella attached to the same body might follow different beating patterns, leading to a complex locomotion strategy that often relies also on the resistance the cell body poses to the fluid. In contrast, propulsion of ciliates derives from the motion of a layer of densely-packed and collectively-moving cilia, which are short hair-like flagella covering their bodies. The seminal review paper of Brennen and Winet (1977) lists a few examples from both groups, highlighting their shape, beat form, geometric characteristics and swimming properties. Cilia may also be used for transport of the surrounding fluid, and their cooperativity can lead to directed flow generation. In higher organisms this can be crucial for internal transport processes, as in cytoplasmic streaming within plant cells (Allen and Allen, 1978), or the transport of ova from the ovary to the uterus in female mammals (Lyons et al., 2006).

Here, we turn our attention to these two morphologically different groups of swimmers to explore the variability of their propulsion dynamics within broad taxonomic groups. To this end, we have collected swimming speed data from literature for flagellated eukaryotes and ciliates and analyze them separately (we do not include spermatozoa since they lack (ironically) the capability to reproduce and are thus not living organisms; their swimming characteristics have been studied by Tam and Hosoi, 2011). A careful examination of the statistical properties of the speed distributions for flagellates and ciliates shows that they are not only both captured by log-normal distributions but that, upon rescaling the data by a characteristic swimming speed for each data set, the speed distributions in both types of organisms are essentially identical.

## Results and discussion

**Figure 1.**
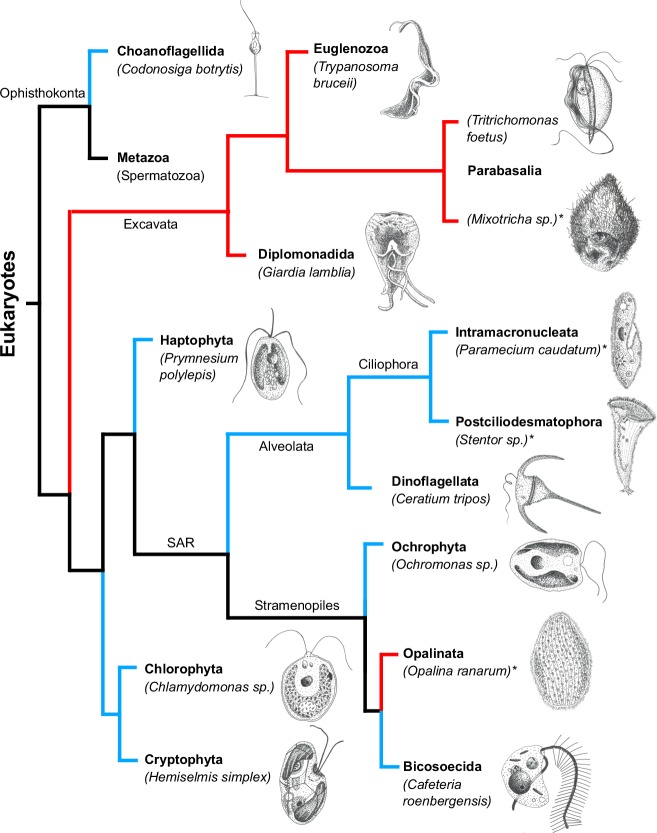
The tree of life (cladogram) for unicellular eukaryotes encompassing the phyla of organisms analyzed in the present study. Aquatic organisms (living in marine, brackish, or freshwater environments) have their branches drawn in blue while parasitic organisms have their branches drawn in red. Ciliates are indicated by an asterisk after their names. For each phylum marked in bold font, a representative organism has been sketched next to its name. Phylogenetic data from Hinchliff et al. (2015).

We have collected swimming data on 189 unicellular eukaryotic microorganisms ($N_{\text{fl}}=112$ flagellates and $N_{\text{cil}}=77$ ciliates) (see Appendix 1 and Source data 1). Figure 1 shows a tree encompassing the phyla of organisms studied and sketches of a representative organism from each phylum. A large morphological variation is clearly visible. In addition, we delineate the branches involving aquatic organisms and parasitic species living within hosts. Both groups include ciliates and flagellates.

**Figure 2.**
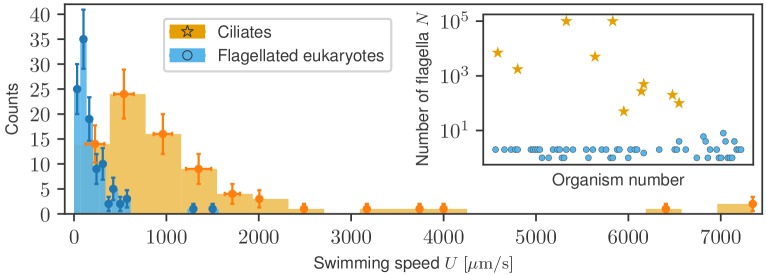
Histograms of swimming speed for ciliates and flagellates demonstrate a similar character but different scales of velocities. Data points represent the mean and standard deviation of the data in each bin; horizontal error bars represent variability within each bin, vertical error bars show the standard deviation of the count. Inset: number of flagella displayed, where available, for each organism exhibits a clear morphological division between ciliates and flagellates.

Due to the morphological and size differences between ciliates and flagellates, we investigate separately the statistical properties of each. Figure 2 shows the two swimming speed histograms superimposed, based on the raw distributions shown in Figure 2—figure supplement 1, where bin widths have been adjusted to their respective samples using the Freedman-Diaconis rule (see Materials and methods). Ciliates span a much larger range of speeds, up to 7 mm/s, whereas generally smaller flagellates remain in the sub-mm/s range. The inset shows that the number of flagella in both groups leads to a clear division. To compare the two groups further, we have also collected information on the characteristic sizes of swimmers from the available literature, which we list in Appendix 1. The average cell size differs fourfold between the populations (31 µm for flagellates and 132 µm for ciliates) and the distributions, plotted in Figure 2—figure supplement 2, are biased towards the low-size end but they are quantitatively different. In order to explore the physical conditions, we used the data on sizes and speeds to compute the Reynolds number Re=U⁢L/ν for each organism, where ν=η/ρ is the kinematic viscosity of water, with η the viscosity and ρ the density. Since almost no data was available for the viscosity of the fluid in swimming speed measurements, we assumed the standard value $\nu =10^{-6}m^{2}/s$ for water for all organisms. The distribution of Reynolds numbers (Figure 2—figure supplement 3), shows that ciliates and flagellates operate in different ranges of Re, although for both groups Re<1, imposing on them the same limitations of inertia-less Stokes flow (Purcell, 1977; Lauga and Powers, 2009).

**Figure 2—figure supplement 1.**
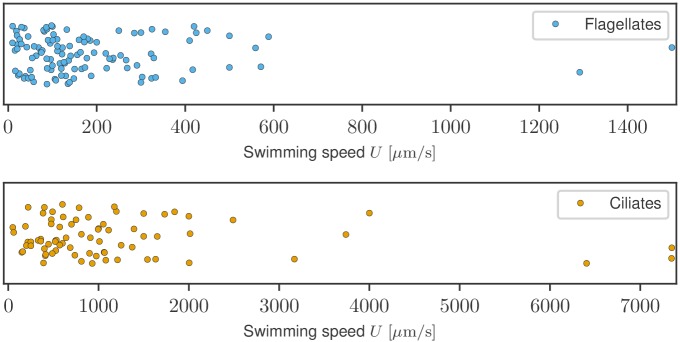
Linear distribution of swimming speed data. Symbols have been randomly placed vertically to avoid overlap.

**Figure 2—figure supplement 2.**
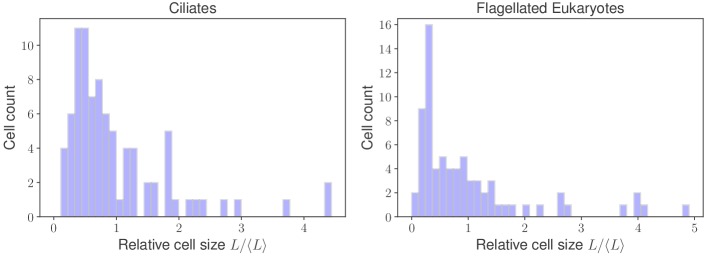
Distribution of organism sizes in analyzed groups. Each histogram has been rescaled by the average cell size for each group. Although both distributions exhibit a qualitatively similar shape biased toward the low limit, no quantitative similarity is found.

**Figure 2—figure supplement 3.**
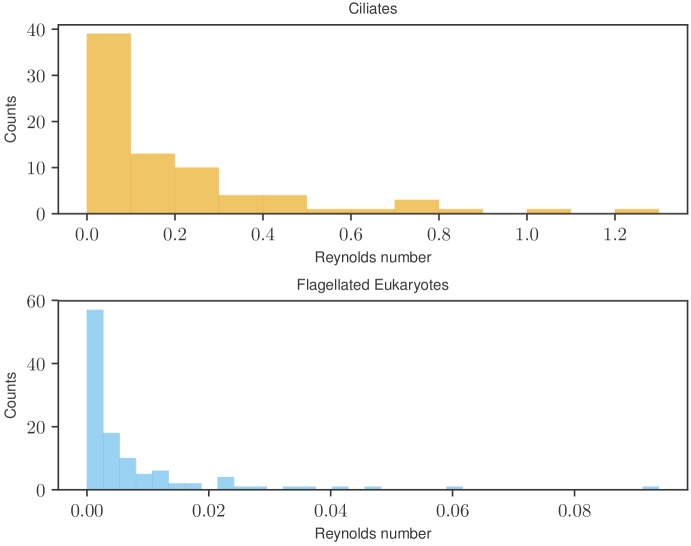
Distribution of Reynolds numbers for organisms in analyzed groups. Source data for the characteristic size L and swimming speeds U are listed in Appendix 1.

Furthermore, studies of green algae (Short et al., 2006; Goldstein, 2015) show that an important distinction between the smaller, flagellated species and the largest multicellular ones involves the relative importance of advection and diffusion, as captured by the Péclet number P⁢e=U⁢L/D, where L is a typical organism size and D is the diffusion constant of a relevant molecular species. Using the average size L of the cell body in each group of the present study ($L_{\text{fl}}=31\;\mu \text{m}$, $L_{\text{cil}}=132\mu \text{m}$) and the median swimming speeds ($U_{\text{fl}}=127m/s$, $U_{cil}=784m/s$), and taking $D=10^{3}(\mu m{)}^{2}/s$, we find $P\,e_{\mathrm{fl}}∼3.9$ and $P\,e_{\mathrm{cil}}∼103$, which further justifies analyzing the groups separately; they live in different physical regimes.

Examination of the mean, variance, kurtosis, and higher moments of the data sets suggest that the probabilities P⁢(U) of the swimming speed are well-described by log-normal distributions,

(1) $$P\,\left(U\right)=\frac{1}{U\,\sigma \,\sqrt{2\,\pi }}\,\exp\left(-\frac{{\left(\ln U-\mu \right)}^{2}}{2\,\sigma^{2}}\right),$$

normalized as $\int_{0}^{\infty }𝑑U\,P\,\left(U\right)=1$, where μ and σ are the mean and the standard deviation of ln⁡U. The median M of the distribution is $e^{\mu }$, with units of speed. Log-normal distributions are widely observed across nature in areas such as ecology, physiology, geology and climate science, serving as an empirical model for complex processes shaping a system with many potentially interacting elements (Limpert et al., 2001), particularly when the underlying processes involve proportionate fluctuations or multiplicative noise (Koch, 1966).

**Figure 3.**
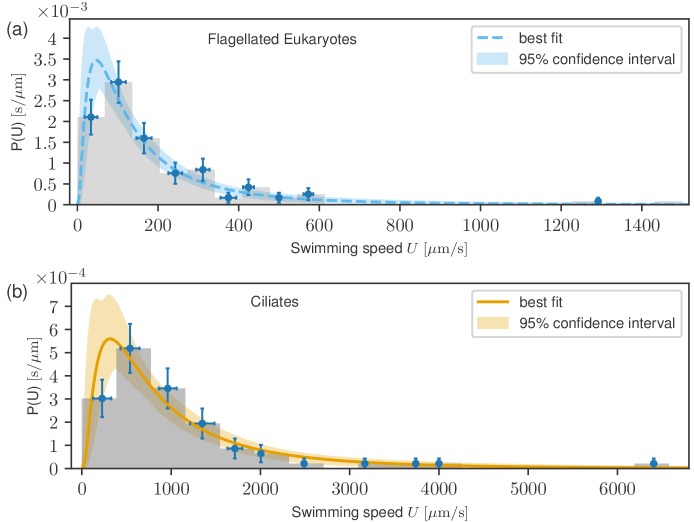
Probability distribution functions of swimming speeds for flagellates (**a**) and ciliates (**b**) with the fitted log-normal distributions. Data points represent uncertainties as in Figure 2. Despite the markedly different scales of the distributions, they have similar shapes.

The results of fitting (see Materials and methods) are plotted in Figure 3, where the best fits are presented as solid curves, with the shaded areas representing 95% confidence intervals. For flagellates, we find the $M_{\text{fl}}=127m/s$ and $\sigma_{\text{fl}}=0.978$ while for ciliates, we obtain $M_{\text{cil}}=784m/s$ and $\sigma_{\text{cil}}=0.936$. Log-normal distributions are known to emerge from an (imperfect) analogy to the Gaussian central limit theorem (see Materials and methods). Since the data are accurately described by this distribution, we conclude that the published literature includes a sufficiently large amount of unbiased data to be able to see the whole distribution.

**Figure 3—figure supplement 1.**
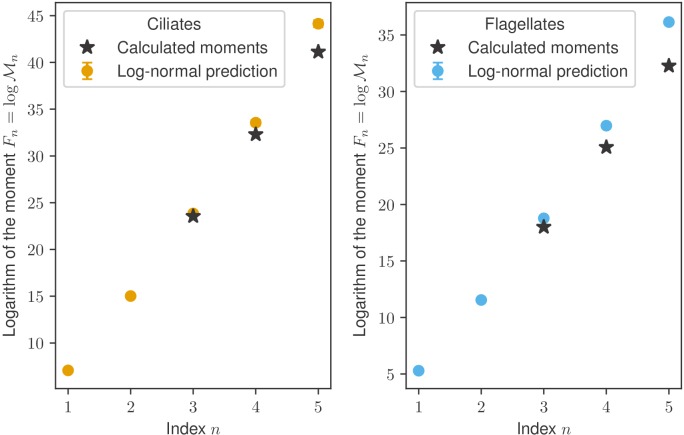
Higher moments of the swimming speed distributions obtained from the data compared with those calculated from the fitted log-normal distribution. The algebraic moments ${ℳ}_{n}$ are defined in Equation (4). Error bars representing 95% confidence intervals for fitted parameters, are obscured by markers.

**Figure 4.**
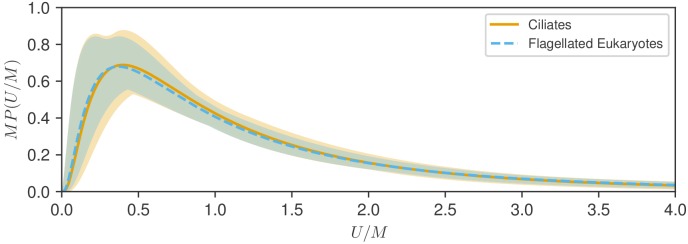
Test of rescaling hypothesis. Shown are the two fitted log-normal curves for flagellates and ciliates, each multiplied by the distribution median M, plotted versus speed normalized by M. The distributions for show remarkable similarity and uncertainty of estimation.

We next compare the statistical variability within groups by examining rescaled distributions (Goldstein, 2018). As each has a characteristic speed M, we align the peaks by plotting the distributions versus the variable U/M for each group. Since P has units of 1/speed, we are thus led to the form $P\,\left(U,M\right)=M^{-1}\,F\,\left(U/M\right)$ for some function F. For the log-normal distribution, with M the median, we find

(2) $$F(\xi )=\frac{1}{\xi \sigma \sqrt{2\pi }}\exp\left(-\frac{\ln^{2}\xi }{2\sigma^{2}}\right),$$

which now depends on the single parameter σ and has a median of unity by construction. To study the similarity of the two distributions we plot the functions F=M⁢P⁢(U/M) for each. As seen in Figure 4, the rescaled distributions are essentially indistinguishable, and this can be traced back to the near identical values of the variances σ, which are within 5% of each other. The fitting uncertainties shown shaded in Figure 4 suggest a very similar range of variability of the fitted distributions. Furthermore, both the integrated absolute difference between the distributions (0.028) and the Kullback-Leibler divergence (0.0016) are very small (see Materials and methods), demonstrating the close similarity of the two distributions. This similarity is robust to the choice of characteristic speed, as shown in Figure 4—figure supplement 1, where the arithmetic mean $U^{*}$ is used in place of the median.

**Figure 4—figure supplement 1.**
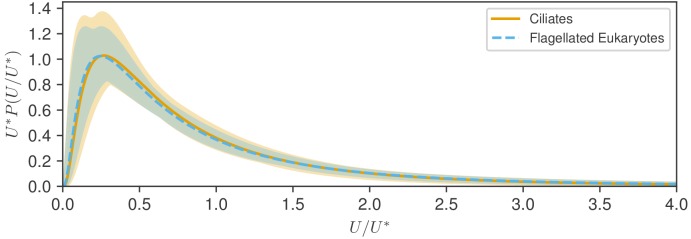
Data collapse as in the main figure, but using the mean speeds $U^{*}$ instead of the median M. A similar quality of data collapse is seen.

In living cells, the sources for intrinsic variability within organisms are well characterized on the molecular and cellular level (Kirkwood et al., 2005) but less is known about variability within taxonomic groups. By dividing unicellular eukaryotes into two major groups on the basis of their difference in morphology, size and swimming strategy, we were able to capture in this paper the log-normal variability within each subset. Using a statistical analysis of the distributions as functions of the median swimming speed for each population we further found an almost identical distribution of swimming speeds for both types of organisms. Our results suggest that the observed log-normal randomness captures a universal way for ecological niches to be populated by abundant microorganisms with similar propulsion characteristics. We note, however, that the distributions of swimming speeds among species do not necessarily reflect the distributions of swimming speeds among individuals, for which we have no available data.

## Materials and methods

### Data collection

Data for ciliates were sourced from 26 research articles, while that for flagellates were extracted from 48 papers (see Appendix 1). Notably, swimming speeds reported in the various studies have been measured under different physiological and environmental conditions, including temperature, viscosity, salinity, oxygenation, pH and light. Therefore we consider the data *not* as representative of a uniform environment, but instead as arising from a random sampling of a wide range of environmental conditions. In cases where no explicit figure was given for U in a paper, estimates were made using other available data where possible. Size of swimmers has also been included as a characteristic length for each organism. This, however, does not reflect the spread and diversity of sizes within populations of individual but is rather an indication of a typical size, as in the considered studies these data were not available. Information on anisotropy (different width/length) is also not included.

No explicit criteria were imposed for the inclusion in the analyses, apart from the biological classification (i.e. whether the organisms were unicellular eukaryotic ciliates/flagellates). We have used all the data found in literature for these organisms over the course of an extensive search. Since no selection was made, we believe that the observed statistical properties are representative for these groups.

### Data processing and fitting the log-normal distribution

Bin widths in histograms in Figure 2 and Figure 3 have been chosen separately for ciliates and flagellated eukaryotes according to the Freedman-Diaconis rule (Freedman and Diaconis, 1981) taking into account the respective sample sizes and the spread of distributions. The bin width b is then given by the number of observations N and the interquartile range of the data IQR as

(3) $$b=2\,\frac{\mathrm{IQR}}{N^{1/3}}.$$

Within each bin in Figure 3, we calculate the mean and the standard deviation for the binned data, which constitute the horizontal error bars. The vertical error bars reflect the uncertainty in the number of counts $N_{j}$ in bin j. This is estimated to be Poissonian, and thus the absolute error amounts to $\sqrt{N_{j}}$. Notably, the relative error decays with the number of counts as $1/\sqrt{N_{j}}$.

In fitting the data, we employ the log-normal distribution Equation (1). In general, from from data comprising N measurements, labelled $x_{i}$ (i=1,…,N), the n-th arithmetic moment ${ℳ}_{n}$ is the expectation $𝔼\,\left(X^{n}\right)$, or

(4) $${ℳ}_{n}=\frac{1}{N}\,\sum_{i=1}^{N}x_{i}^{n}$$

Medians of the data were found by sorting the list of values and picking the middlemost value. For a log-normal distribution, the arithmetic moments are given solely by μ and σ of the associated normal distribution as

(5) $${ℳ}_{n}=M^{n}\,\Sigma^{n^{2}},$$

where we have defined M=exp⁡(μ) and $\Sigma =\exp\left(\sigma^{2}/2\right)$, and note that M is the median of the distribution. Thus, the mean is M⁢Σ and the variance is $M^{2}\,\Sigma^{2}\,\left(\Sigma^{2}-1\right)$. From the first and second moments, we estimate

(6) $$\mu =\ln\left(\frac{{ℳ}_{1}^{2}}{\sqrt{{ℳ}_{2}}}\right)\mathrm{and}\sigma^{2}=\ln\left(\frac{{ℳ}_{2}}{{ℳ}_{1}^{2}}\right).$$

Having estimated μ and σ, we can compute the higher order moments from Equation (5) and compare to those calculated directly from the data, as shown in Figure 3—figure supplement 1.

To fit the data, we have used both the MATLAB fitting routines and the Python scipy.stats module. From these fits we estimated the shape and scale parameters and the 95% confidence intervals in Figure 3 and Figure 4. We emphasize that the fitting procedures use the raw data via the maximum likelihood estimation method, and not the processed histograms, hence the estimated parameters are insensitive to the binning procedure.

For rescaled distributions, the average velocity for each group of organisms was calculated as $U^{*}=\frac{1}{N_{i}}\,\sum_{i=1}^{N_{i}}U_{i}$, with i∈{cil,fl}. Then, data in each subset have been rescaled by the area under the fitted curve to ensure that the resulting probability density functions $p_{i}$ are normalized as

(7) $$\int_{0}^{\infty }p_{i}\,\left(x\right)\,dx=1.$$

In characterizations of biological or ecological diversity, it is often assumed that the examined variables are Gaussian, and thus the distribution of many uncorrelated variables attains the normal distribution by virtue of the Central Limit Theorem (CLT). In the case when random variables in question are positive and have a log-normal distribution, no analogous explicit analytic result is available. Despite that, there is general agreement that a sum of independent log-normal random variables can be well approximated by another log-normal random variable. It has been proven by Szyszkowicz and Yanikome (2009) that the sum of identically distributed equally and positively correlated joint log-normal distributions converges to a log-normal distribution of known characteristics but for uncorrelated variables only estimations are available (Beaulieu et al., 1995). We use these results to conclude that our distributions contain enough data to be unbiased and seen in full.

### Comparisons of distributions

In order to quantify the differences between the fitted distributions, we define the integrated absolute difference Δ between two probability distributions p⁢(x) and q⁢(x) (x>0) as

(8) $$\Delta =\int_{0}^{\infty }\left|p\,\left(x\right)-q\,\left(x\right)\right|\,dx.$$

As the probability distributions are normalized, this is a measure of their relative ’distance’. As a second measure, we use the Kullback-Leibler divergence (Kullback and Leibler, 1951),

(9) $$D\,\left(p,q\right)=\int_{0}^{\infty }p\,\left(x\right)\,\ln\left(\frac{p\,\left(x\right)}{q\,\left(x\right)}\right)\,dx.$$

Note that D⁢(p,q)≠D⁢(q,p) and therefore D is not a distance metric in the space of probability distributions.

## Data Availability

All data generated or analysed during this study are included in the manuscript.

## Additional files

Source data 1. Spreadsheet data for swimming eukaryotes listed in Appendix 1 and Appendix 2.

Transparent reporting form

## Appendix 1

10.7554/eLife.44907.013 The Appendix contains the data which form the basis of our study. The tables contain data on the sizes and swimming speed of ciliates organisms and flagellated eukaryotes from the existing literature. Data for ciliates were sourced from 26 research articles, while data for the flagellates were extracted from 48 papers. In the cases where two or more sources reported contrasting figures for the swimming speed, the average value is reported in our tables. The data itself is available in Source data 1. Data for swimming flagellates Abbreviations: dflg. – dinoflagellata; dph – dinophyceae; chlph. – chlorophyta; ochph. (het.) –ochrophyta (heterokont); srcm. – sarcomastigophora, pyr. – pyramimonadophyceae; prym. – prymnesiophyceae; dict. – dictyochophyceae; crypt. – cryptophyceae; chrys. – chrysophyceae **Table:**

Species	Phylum	Class	L[μ⁢m]	U[μ⁢m/s]	References
*Alexandrium minutum*	dflg.	dph.	21.7	222.5	(Lewis et al., 2006)
*Alexandrium ostenfeldii*	dflg.	dph.	41.1	110.5	(Lewis et al., 2006)
*Alexandrium tamarense*	dflg.	dph.	26.7	200	(Lewis et al., 2006)
*Amphidinium britannicum*	dflg.	dph.	51.2	68.7	(Bauerfeind et al., 1986)
*Amphidinium carterae*	dflg.	dph.	16	81.55	(Gittleson et al., 1974; Bauerfeind et al., 1986)
*Amphidinium klebsi*	dflg.	dph.	35	73.9	(Gittleson et al., 1974)
*Apedinella spinifera*	ochph. (het.)	dict.	8.25	132.5	(Throndsen, 1973)
*Bodo designis*	euglenozoa	kinetoplastea	5.5	39	(Visser and Kiørboe, 2006)
*Brachiomonas submarina*	chlph.	chlorophyceae	27.5	96	(Bauerfeind et al., 1986)
*Cachonina (Heterocapsa) niei*	dflg.	dph.	21.4	302.8	(Levandowsky and Kaneta, 1987; Kamykowski and Zentara, 1977)
*Cafeteria roenbergensis*	bygira (heterokont)	bicosoecida	2	94.9	(Fenchel and Blackburn, 1999)
*Ceratium cornutum*	dflg.	dph.	122.3	177.75	(Levandowsky and Kaneta, 1987; Metzner, 1929)
*Ceratium furca*	dflg.	dph.	122.5	194	(Peters, 1929)
*Ceratium fusus*	dflg.	dph.	307.5	156.25	(Peters, 1929)
*Ceratium hirundinella*	dflg.	dph.	397.5	236.1	(Levandowsky and Kaneta, 1987)
*Ceratium horridum*	dflg.	dph.	225	20.8	(Peters, 1929)
*Ceratium lineatus*	dflg.	dph.	82.1	36	(Fenchel, 2001)
*Ceratium longipes*	dflg.	dph.	210	166	(Peters, 1929)
*Ceratium macroceros*	dflg.	dph.	50	15.4	(Peters, 1929)
*Ceratium tripos*	dflg.	dph.	152.3	121.7	(Peters, 1929; Bauerfeind et al., 1986)
*Chilomonas paramecium*	cryptophyta	crypt.	30	111.25	(Lee, 1954; Jahn and Bovee, 1967; Gittleson et al., 1974)
*Chlamydomonas reinhardtii*	chlph.	chlorophyceae	10	130	(Gittleson et al., 1974; Roberts, 1981; Guasto et al., 2010)
*Chlamydomonas moewusii*	chlph.	chlorophyceae	12.5	128	(Gittleson et al., 1974)
*Chlamydomonas sp.*	chlph.	chlorophyceae	13	63.2	(Lowndes, 1944; Lowndes, 1941; Bauerfeind et al., 1986)
*Crithidia deanei*	euglenozoa	kinetoplastea	7.4	45.6	(Gadelha et al., 2007)
*Crithidia fasciculata*	euglenozoa	kinetoplastea	11.1	54.3	(Gadelha et al., 2007)
*Crithidia (Strigomonas) oncopelti*	euglenozoa	kinetoplastea	8 .1	18.5	(Roberts, 1981; Gittleson et al., 1974)
*Crypthecodinium cohnii*	dflg.	dph.	n/a	122.8	(Fenchel, 2001)
*Dinophysis acuta*	dflg.	dph.	65	500	(Peters, 1929)
*Dinophysis ovum*	dflg.	dph.	45	160	(Buskey et al., 1993)
*Dunaliella* sp.	chlph.	chlorophyceae	10.8	173.5	(Gittleson et al., 1974; Bauerfeind et al., 1986)
*Euglena gracilis*	euglenozoa	euglenida (eugl.)	47.5	111.25	(Lee, 1954; Jahn and Bovee, 1967; Gittleson et al., 1974)
*Euglena viridis*	euglenozoa	euglenida (eugl.)	58	80	(Holwill, 1975; Roberts, 1981; Lowndes, 1941)
*Eutreptiella gymnastica*	euglenozoa	euglenida (aphagea)	23.5	237.5	(Throndsen, 1973)
*Eutreptiella* sp. R	euglenozoa	euglenida	50	135	(Throndsen, 1973)
*Exuviaella baltica (Prorocentrum balticum)*	dflg.	dph.	15.5	138.9	(Wheeler, 1966)
*Giardia lamblia*	srcm.	zoomastigophora	11.25	26	(Lenaghan et al., 2011; Campanati et al., 2002; Chen et al., 2012)
*Gonyaulax polyedra*	dflg.	dph.	39.2	254.05	(Hand et al., 1965; Gittleson et al., 1974; Kamykowski et al., 1992)
*Gonyaulax polygramma*	dflg.	dph.	46.2	500	(Levandowsky and Kaneta, 1987)
*Gymnodinium aureolum*	dflg.	dph.	n/a	394	(Meunier et al., 2013)
*Gymnodinium sanguineum (splendens)*	dflg.	dph.	47.6	220.5	(Kamykowski et al., 1992; Levandowsky and Kaneta, 1987)
*Gymnodinium simplex*	dflg.	dph.	10.6	559	(Jakobsen et al., 2006)
*Gyrodinium aureolum*	dflg.	dph.	30.5	139	(Bauerfeind et al., 1986; Throndsen, 1973)
*Gyrodinium dorsum* (bi-flagellated)	dflg.	dph.	37.5	324	(Hand et al., 1965; Gittleson et al., 1974; Kamykowski et al., 1992; Levandowsky and Kaneta, 1987; Brennen and Winet, 1977)
*Gyrodinium dorsum* (uni-flagellated)	dflg.	dph.	34.5	148.35	(Hand and Schmidt, 1975)
*Hemidinium nasutum*	dflg.	dph.	27.2	105.6	(Levandowsky and Kaneta, 1987; Metzner, 1929)
*Hemiselmis simplex*	cryptophyta	crypt.	5.25	325	(Throndsen, 1973)
*Heterocapsa pygmea*	dflg.	dph.	13.5	102.35	(Bauerfeind et al., 1986)
*Heterocapsa rotundata*	dflg.	dph.	12.5	323	(Jakobsen et al., 2006)
*Heterocapsa triquetra*	dflg.	dph.	17	97	(Visser and Kiørboe, 2006)
*Heteromastix pyriformis*	chlph.	nephrophyseae	6	87.5	(Throndsen, 1973)
*Hymenomonas carterae*	haptophyta	prym.	12.5	87	(Bauerfeind et al., 1986)
*Katodinium rotundatum (Heterocapsa rotundata)*	dflg.	dph.	10.8	425	(Levandowsky and Kaneta, 1987; Throndsen, 1973)
*Leishmania major*	euglenozoa	kinetoplastea	12.5	36.4	(Gadelha et al., 2007)
*Menoidium cultellus*	euglenozoa	euglenida (eugl.)	45	136.75	(Holwill, 1975; Votta et al., 1971)
*Menoidium incurvum*	euglenozoa	euglenida (eugl.)	25	50	(Lowndes, 1941; Gittleson et al., 1974)
*Micromonas pusilla*	chlph.	mamiellophyceae	2	58.5	(Bauerfeind et al., 1986; Throndsen, 1973)
*Monas stigmata*	ochph. (het.)	chrys.	6	269	(Gittleson et al., 1974)
*Monostroma angicava*	chlph.	ulvophyceae	6.7	170.55	(Togashi et al., 1997)
*Nephroselmis pyriformis*	chlph.	nephrophyseae	4.8	163.5	(Bauerfeind et al., 1986)
*Oblea rotunda*	dflg.	dph.	20	420	(Buskey et al., 1993)
*Ochromonas danica*	ochph. (het.)	chrys.	8.7	77	(Holwill and Peters, 1974)
*Ochromonas malhamensis*	ochph. (het.)	chrys.	3	57.5	(Holwill, 1974)
*Ochromonas minima*	ochph. (het.)	chrys.	5	75	(Throndsen, 1973)
*Olisthodiscus luteus*	ochph. (het.)	raphidophyceae	22.5	90	(Bauerfeind et al., 1986; Throndsen, 1973)
*Oxyrrhis marina*	dflg.	oxyrrhea	39.5	300	(Boakes et al., 2011; Fenchel, 2001)
*Paragymnodinium shiwhaense*	dflg.	dph.	10.9	571	(Meunier et al., 2013)
*Paraphysomonas vestita*	ochph. (het.)	chrys.	14.7	116.85	(Christensen-Dalsgaard and Fenchel, 2004)
*Pavlova lutheri*	haptophyta	pavlovophyceae	6.5	126	(Bauerfeind et al., 1986)
*Peranema trichophorum*	euglenozoa	euglenida (heteronematales)	45	20	(Lowndes, 1941; Gittleson et al., 1974; Brennen and Winet, 1977)
*Peridinium bipes*	dflg.	dph.	42.9	291	(Fenchel, 2001)
*Peridinium cf. quinquecorne*	dflg.	dph.	19	1500	(Bauerfeind et al., 1986; Levandowsky and Kaneta, 1987; Horstmann, 1980)
*Peridinium cinctum*	dflg.	dph.	47.5	120	(Bauerfeind et al., 1986; Levandowsky and Kaneta, 1987; Metzner, 1929)
*Peridinium (Protoperidinium) claudicans*	dflg.	dph.	77.5	229	(Peters, 1929)
*Peridinium (Protoperidinium) crassipes*	dflg.	dph.	102	100	(Peters, 1929)
*Peridinium foliaceum*	dflg.	dph.	30.6	185.2	(Kamykowski et al., 1992)
*Peridinium (Bysmatrum) gregarium*	dflg.	dph.	32.5	1291.7	(Levandowsky and Kaneta, 1987)
*Peridinium (Protoperidinium) ovatum*	dflg.	dph.	61	187.5	(Peters, 1929)
*Peridinium (Peridiniopsis) penardii*	dflg.	dph.	28.8	417	(Sibley et al., 1974)
*Peridinium (Protoperidinium) pentagonum*	dflg.	dph.	92.5	266.5	(Peters, 1929)
*Peridinium (Protoperidinium) subinerme*	dflg.	dph.	50	285	(Peters, 1929)
*Peridinium trochoideum*	dflg.	dph.	25	53	(Levandowsky and Kaneta, 1987)
*Peridinium umbonatum*	dflg.	dph.	30	250	(Levandowsky and Kaneta, 1987; Metzner, 1929)
*Phaeocystis pouchetii*	haptophyta	prym.	6.3	88	(Bauerfeind et al., 1986)
*Polytoma uvella*	chlph.	chlorophyceae	22.5	100.9	(Lowndes, 1944; Gittleson et al., 1974; Lowndes, 1941)
*Polytomella agilis*	chlph.	chlorophyceae	12.4	150	(Gittleson and Jahn, 1968; Gittleson and Noble, 1973; Gittleson et al., 1974; Roberts, 1981)
*Prorocentrum mariae-lebouriae*	dflg.	dph.	14.8	141.05	(Kamykowski et al., 1992; Bauerfeind et al., 1986; Miyasaka et al., 1998)
*Prorocentrum micans*	dflg.	dph.	45	329.1	(Bauerfeind et al., 1986; Levandowsky and Kaneta, 1987)
*Prorocentrum minimum*	dflg.	dph.	15.1	107.7	(Bauerfeind et al., 1986; Miyasaka et al., 1998)
*Prorocentrum redfieldii Bursa (P.triestinum)*	dflg.	dph.	33.2	333.3	(Sournia, 1982)
*Protoperidinium depressum*	dflg.	dph.	132	450	(Buskey et al., 1993)
*Protoperidinium granii (Ostf.) Balech*	dflg.	dph.	57.5	86.1	(Sournia, 1982)
*Protoperidinium pacificum*	dflg.	dph.	54	410	(Buskey et al., 1993)
*Prymnesium polylepis*	haptophyta	prym.	9.1	45	(Dölger et al., 2017)
*Prymnesium parvum*	haptophyta	prym.	7.2	30	(Dölger et al., 2017)
*Pseudopedinella pyriformis*	ochph. (het.)	dict.	6.5	100	(Throndsen, 1973)
*Pseudoscourfieldia marina*	chlph.	pyr.	4.1	42	(Bauerfeind et al., 1986)
*Pteridomonas danica*	ochph. (het.)	dict.	5.5	179.45	(Christensen-Dalsgaard and Fenchel, 2004)
*Pyramimonas amylifera*	chlph.	pyr.	24.5	22.5	(Bauerfeind et al., 1986)
*Pyramimonas cf. disomata*	chlph.	pyr.	9	355	(Throndsen, 1973)
*Rhabdomonas spiralis*	euglenozoa	euglenida (aphagea)	27	120	(Holwill, 1975)
*Rhodomonas salina*	cryptophyta	crypt.	14.5	588.5	(Jakobsen et al., 2006; Meunier et al., 2013)
*Scrippsiella trochoidea*	dflg.	dph.	25.3	87.6	(Kamykowski et al., 1992; Bauerfeind et al., 1986; Sournia, 1982)
*Spumella* sp.	ochph. (het.)	chrys.	10	25	(Visser and Kiørboe, 2006)
*Teleaulax* sp.	cryptophyta	crypt.	13.5	98	(Meunier et al., 2013)
*Trypanosoma brucei*	euglenozoa	kinetoplastea	18.8	20.5	(Rodríguez et al., 2009)
*Trypanosoma cruzi*	euglenozoa	kinetoplastea	20	172	(Jahn and Fonseca, 1963; Brennen and Winet, 1977)
*Trypanosoma vivax*	euglenozoa	kinetoplastea	23.5	29.5	(Bargul et al., 2016)
*Trypanosoma evansi*	euglenozoa	kinetoplastea	21.5	16.1	(Bargul et al., 2016)
*Trypanosoma congolense*	euglenozoa	kinetoplastea	18	9.7	(Bargul et al., 2016)
*Tetraflagellochloris mauritanica*	chlph.	chlorophyceae	4	300	(Barsanti et al., 2016)

## Appendix 2

10.7554/eLife.44907.014 Data for swimming ciliates Abbreviations: imnc. = intramacronucleata; pcdph. = postciliodesmatophora; olig. – oligohymenophorea; spir. – spirotrichea; hettr. – heterotrichea; lit. – litostomatea; eugl. – euglenophyceae **Table:**

Species	Phylum	Class	L[μ⁢m]	U[μ⁢m/s]	References
*Amphileptus gigas*	imnc.	lit.	808	608	(Bullington, 1925)
*Amphorides quadrilineata*	imnc.	spir.	138	490	(Buskey et al., 1993)
*Balanion comatum*	imnc.	prostomatea	16	220	(Visser and Kiørboe, 2006)
*Blepharisma*	pcdph.	hettr.	350	600	(Sleigh and Blake, 1977; Roberts, 1981)
*Coleps hirtus*	imnc.	prostomatea	94.5	686	(Bullington, 1925)
*Coleps* sp.	imnc.	prostomatea	78	523	(Bullington, 1925)
*Colpidium striatum*	imnc.	olig.	77	570	(Beveridge et al., 2010)
*Condylostoma patens*	pcdph.	hettr.	371	1061	(Bullington, 1925; Machemer, 1974)
*Didinium nasutum*	imnc.	lit.	140	1732	(Bullington, 1925; Machemer, 1974; Roberts, 1981; Sleigh and Blake, 1977)
*Euplotes charon*	imnc.	spir.	66	1053	(Bullington, 1925)
*Euplotes patella*	imnc.	spir.	202	1250	(Bullington, 1925)
*Euplotes vannus*	imnc.	spir.	82	446	(Wang et al., 2008; Ricci et al., 1997)
*Eutintinnus cf. pinguis*	imnc.	spir.	147	410	(Buskey et al., 1993)
*Fabrea salina*	pcdph.	hettr.	184.1	216	(Marangoni et al., 1995)
*Favella panamensis*	imnc.	spir.	238	600	(Buskey et al., 1993)
*Favella* sp.	imnc.	spir.	150	1080	(Buskey et al., 1993)
*Frontonia* sp.	imnc.	olig.	378.5	1632	(Bullington, 1925)
*Halteria grandinella*	imnc.	spir.	50	533	(Bullington, 1925; Gilbert, 1994)
*Kerona polyporum*	imnc.	spir.	107	476.5	(Bullington, 1925)
*Laboea strobila*	imnc.	spir.	100	810	(Buskey et al., 1993)
*Lacrymaria lagenula*	imnc.	lit.	45	909	(Bullington, 1925)
*Lembadion bullinum*	imnc.	olig.	43	415	(Bullington, 1925)
*Lembus velifer*	imnc.	olig.	87	200	(Bullington, 1925)
*Mesodinium rubrum*	imnc.	lit.	38	7350	(Jonsson and Tiselius, 1990; Riisgård and Larsen, 2009; Crawford and Lindholm, 1997)
*Metopides contorta*	imnc.	armophorea	115	359	(Bullington, 1925)
*Nassula ambigua*	imnc.	nassophorea	143	2004	(Bullington, 1925)
*Nassula ornata*	imnc.	nassophorea	282	750	(Bullington, 1925)
*Opalina ranarum*	placidozoa (heterokont)	opalinea	350	50	(Blake, 1975; Sleigh and Blake, 1977)
*Ophryoglena* sp.	imnc.	olig.	325	4000	(Machemer, 1974)
*Opisthonecta henneg*	imnc.	olig.	126	1197	(Machemer, 1974; Jahn and Hendrix, 1969)
*Oxytricha bifara*	imnc.	spir.	282	1210	(Bullington, 1925)
*Oxytricha ferruginea*	imnc.	spir.	150	400	(Bullington, 1925)
*Oxytricha platystoma*	imnc.	spir.	130	520	(Bullington, 1925)
*Paramecium aurelia*	imnc.	olig.	244	1650	(Bullington, 1925; Bullington, 1930)
*Paramecium bursaria*	imnc.	olig.	130	1541.5	(Bullington, 1925; Bullington, 1930)
*Paramecium calkinsii*	imnc.	olig.	124	1392	(Bullington, 1930; Bullington, 1925)
*Paramecium caudatum*	imnc.	olig.	225.5	2489.35	(Bullington, 1930; Jung et al., 2014)
*Paramecium marinum*	imnc.	olig.	115	930	(Bullington, 1925)
*Paramecium multimicronucleatum*	imnc.	olig.	251	3169.5	(Bullington, 1930)
*Paramecium polycaryum*	imnc.	olig.	91	1500	(Bullington, 1930)
*Paramecium* spp.	imnc.	olig.	200	975	(Jahn and Bovee, 1967; Sleigh and Blake, 1977; Roberts, 1981)
*Paramecium tetraurelia*	imnc.	olig.	124	784	(Funfak et al., 2015)
*Paramecium woodruffi*	imnc.	olig.	160	2013.5	(Bullington, 1930)
*Porpostoma notatum*	imnc.	olig.	107.7	1842.2	(Fenchel and Blackburn, 1999)
*Prorodon teres*	imnc.	prostomatea	175	1066	(Bullington, 1925)
*Spathidium spathula*	imnc.	lit.	204.5	526	(Bullington, 1925)
*Spirostomum ambiguum*	pcdph.	hettr.	1045	810	(Bullington, 1925)
*Spirostomum* sp.	pcdph.	hettr.	1000	1000	(Sleigh and Blake, 1977)
*Spirostomum teres*	pcdph.	hettr.	450	640	(Bullington, 1925)
*Stenosemella steinii*	imnc.	spir.	83	190	(Buskey et al., 1993)
*Stentor caeruleus*	pcdph.	hettr.	528.5	1500	(Bullington, 1925)
*Stentor polymorphus*	pcdph.	hettr.	208	887	(Bullington, 1925; Sleigh and Aiello, 1972; Sleigh, 1968)
*Strobilidium spiralis*	imnc.	spir.	60	330	(Buskey et al., 1993)
*Strobilidium velox*	imnc.	spir.	43	150	(Gilbert, 1994)
*Strombidinopsis acuminatum*	imnc.	spir.	80	390	(Buskey et al., 1993)
*Strombidium claparedi*	imnc.	spir.	69.5	3740	(Bullington, 1925)
*Strombidium conicum*	imnc.	spir.	75	570	(Buskey et al., 1993)
*Strombidium* sp.	imnc.	spir.	33	360	(Buskey et al., 1993)
*Strombidium sulcatum*	imnc.	spir.	32.5	995	(Fenchel and Jonsson, 1988; Fenchel and Blackburn, 1999 Fenchel and Blackburn, 1999)
*Stylonichia* sp.	imnc.	spir.	167	737.5	(Bullington, 1925; Machemer, 1974)
*Tetrahymena pyriformis*	imnc.	olig.	72.8	475.6	(Sleigh and Blake, 1977; Roberts, 1981; Brennen and Winet, 1977)
*Tetrahymena thermophila*	imnc.	olig.	46.7	204.5	(Wood et al., 2007)
*Tillina magna*	imnc.	colpodea	162.5	2000	(Bullington, 1925)
*Tintinnopsis kofoidi*	imnc.	spir.	100	400	(Buskey et al., 1993)
*Tintinnopsis minuta*	imnc.	spir.	40	60	(Buskey et al., 1993)
*Tintinnopsis tubulosa*	imnc.	spir.	95	160	(Buskey et al., 1993)
*Tintinnopsis vasculum*	imnc.	spir.	82	250	(Buskey et al., 1993)
*Trachelocerca olor*	pcdph.	karyorelictea	267.5	900	(Bullington, 1925)
*Trachelocerca tenuicollis*	pcdph.	karyorelictea	432	1111	(Bullington, 1925)
*Uroleptus piscis*	imnc.	spir.	203	487	(Bullington, 1925)
*Uroleptus rattulus*	imnc.	spir.	400	385	(Bullington, 1925)
*Urocentrum turbo*	imnc.	olig.	90	700	(Bullington, 1925)
*Uronema filificum*	imnc.	olig.	25.7	1372.7	(Fenchel and Blackburn, 1999)
*Uronema marinum*	imnc.	olig.	56.9	1010	(Fenchel and Blackburn, 1999)
*Uronema sp.*	imnc.	olig.	25	1175	(Sleigh and Blake, 1977; Roberts, 1981)
*Uronychia transfuga*	imnc.	spir.	118	6406	(Leonildi et al., 1998)
*Uronychia setigera*	imnc.	spir.	64	7347	(Leonildi et al., 1998)
*Uronemella* spp.	imnc.	olig.	28	250	(Petroff et al., 2015)
